# The ATP-Dependent Protease ClpP Inhibits Biofilm Formation by Regulating Agr and Cell Wall Hydrolase Sle1 in *Staphylococcus aureus*

**DOI:** 10.3389/fcimb.2017.00181

**Published:** 2017-05-15

**Authors:** Qian Liu, Xing Wang, Juanxiu Qin, Sen Cheng, Won-Sik Yeo, Lei He, Xiaowei Ma, Xiaoyun Liu, Min Li, Taeok Bae

**Affiliations:** ^1^Department of Laboratory Medicine, School of Medicine, Ren Ji Hospital, Shanghai Jiao Tong UniversityShanghai, China; ^2^Department of Laboratory Medicine, Shanghai Children's Medical Center, School of Medicine, Shanghai Jiao Tong UniversityShanghai, China; ^3^Institute of Analytical Chemistry and Synthetic and Functional Biomolecules Center, College of Chemistry and Molecular Engineering, Peking UniversityBeijing, China; ^4^Department of Microbiology and Immunology, Indiana University School of Medicine-NorthwestGary, IN, USA

**Keywords:** *Staphylococcus aureus*, biofilm, proteolysis system, Agr, cell wall hydrolysis

## Abstract

Biofilm causes hospital-associated infections on indwelling medical devices. In *Staphylococcus aureus*, Biofilm formation is controlled by intricately coordinated network of regulating systems, of which the ATP-dependent protease ClpP shows an inhibitory effect. Here, we demonstrate that the inhibitory effect of ClpP on biofilm formation is through Agr and the cell wall hydrolase Sle1. Biofilm formed by *clpP* mutant consists of proteins and extracellular DNA (eDNA). The increase of the protein was, at least in part, due to the reduced protease activity of the mutant, which was caused by the decreased activity of *agr*. On the other hand, the increase of eDNA was due to increased cell lysis caused by the higher level of Sle1. Indeed, as compared with wild type, the *clpP* mutant excreted an increased level of eDNA, and showed higher sensitivity to Triton-induced autolysis. The deletion of *sle1* in the *clpP* mutant decreased the biofilm formation, the level of eDNA, and the Triton-induced autolysis to wild-type levels. Despite the increased biofilm formation capability, however, the *clpP* mutant showed significantly reduced virulence in a murine model of subcutaneous foreign body infection, indicating that the increased biofilm formation capability cannot compensate for the intrinsic functions of ClpP during infection.

## Introduction

Bacteria biofilms are complex communities which have intrinsic resistance to host immune defenses and antibiotic treatment (Paharik and Horswill, 2016). In the important human pathogen *Staphylococcus aureus*, biofilm enables the bacterium to persist during infections on indwelling medical devices, endocarditis, or chronic wound infections (Otto, 2013). Depending on its constituents, staphylococcal biofilm can be polysaccharide intercellular adhesion (PIA)-dependent or proteins/ extracellular DNA (eDNA)-dependent (McCarthy et al., 2015). PIA is synthesized by the enzyme encoded by the *icaADBC* operon, and can be an important component of the biofilm matrix (Cramton et al., 1999; Gotz, 2002; Jefferson et al., 2004). However, several studies have shown that *S. aureus* can form a biofilm by proteins and eDNA without PIA-involvement (Toledo-Arana et al., 2005; Rohde et al., 2007). For proteins, protein A (Spa), and the fibronectin-binding proteins FnbpA and FnbpB, are known to play an important role in the biofilm matrix formation (O'Neill et al., 2008; Merino et al., 2009). In the PIA-independent biofilm, extracellular DNA (eDNA) is also an important component of biofilm matrix (Kiedrowski et al., 2011; Okshevsky and Meyer, 2015). For example, secreted protease Esp inhibits biofilm formation by cleaving murein hydrolase autolysin (Atl) and preventing release of eDNA (Chen et al., 2013).

Biofilm formation in *S. aureus* is controlled by an intricate network of regulatory systems. Rot (repressor of toxin) contributes to biofilm formation by down-regulating secreted proteases (Mootz et al., 2015). The accessory gene regulator (Agr) is known to inhibit biofilm formation by up-regulating extracellular proteases, an important contributor to the dispersal of established biofilm (Boles and Horswill, 2008). Sigma factor B (*sigB*) contributes to biofilm formation by suppressing the expression of RNAIII, the effector molecule of Agr, which, in turn, leads to decreased levels of extracellular proteases (Lauderdale et al., 2009; Marti et al., 2010). Recently, AraC-type transcriptional regulator, Rsp, was shown to inhibit biofilm formation by repressing the expression of biofilm-associated genes such as *ica* operon (Li et al., 2015).

Another important biofilm regulatory system is the ATP-dependent protease ClpP, the proteolytic subunit of Clp proteases (Arnold and Langer, 2002; Dalbey et al., 2012). In Clp proteases, the proteolytic chamber is formed by two hexameric rings of ClpP subunits, whereas the ATPase function is provided by Clp ATPases such as ClpB, ClpC, ClpL, and ClpX. The ClpP proteolytic activity is reported to play a critical role on virulence, stress response, and physiology in *S. aureus* (Frees et al., 2004). In the *S. aureus* 8325-4 strain, while ATPases ClpX and ClpC promote biofilm formation, ClpP represses it (Frees et al., 2004). However, it is not known whether the biofilm inhibitory effect of ClpP is universal, and, if so, how ClpP does it.

In this study, we found that the biofilm inhibitory effect of ClpP is conserved among different *S. aureus* strains. Moreover, we further demonstrate that the inhibitory effect is, at least in part, due to ClpP's effect on the quorum sensing system Agr, and the cell wall hydrolyzing enzyme Sle1.

## Materials and methods

### Ethics statement

All animal experiments protocols were performed following the Guide for the Care and Use of Laboratory Animals of the Chinese Association for Laboratory Animal Sciences (CALAS) and were approved by the ethics committee of Renji Hospital, School of medicine, Shanghai Jiaotong University, Shanghai, China.

### Bacterial strains, plasmids and culture conditions

All experiments were performed with *S. aureus* Newman (NM) or USA300 as the wild-type strains. The bacterial strains and plasmids used in this study are listed in Supplementary Table 1. *Escherichia coli* and *S. aureus* were grown in Luria-Bertani (LB) broth and tryptic soy broth (TSB), respectively. However, for transduction of plasmids, heart infusion broth (HIB) supplemented with 5 mM CaCl_2_ was used. When necessary, antibiotics were added to the growth media at the following concentrations: ampicillin, 100 μg/ml; erythromycin, 10 μg/ml; and chloramphenicol, 5 μg/ml.

### Construction of plasmids

To construct the plasmid for deleting the *lytM, sle1*, or *agr* genes in Newman and USA300 strains, we used a ligation independent cloning (LIC) method (Aslanidis and De Jong, 1990). First, vector DNA was PCR-amplified from pKOR1 using the primers P236/237 (Supplementary Table 2). One- kb DNA fragments, upstream and downstream of *lytM, sle1*, or *agr*, were amplified by PCR from the chromosomal DNA with the primer pairs P596/597 and P598/599 (for *lytM*), P632/633, and P634/637 (for *sle1*) or P19/20 and P21/22 (for *agr*) (Supplementary Table 2) and PrimSTAR (Takara), a PCR enzyme with high fidelity. The PCR products were treated with T4 DNA polymerase in the presence of dCTP (vector) or dGTP (insert DNA) and mixed together. The DNA mixture was used to transform *E. coli* DH5α. The resulting plasmids, pKOR1Δ*lytM*, pKOR1Δ*sle1*, or pKOR1Δ*agr* were electroporated into *S. aureus* strain RN4220 and subsequently transduced into NM or USA300 with Φ85. The deletion was carried out as described previously (Bae and Schneewind, 2006).

To generate complement plasmids for *clpP* mutant strain, First, vector DNA was PCR-amplified from pCL55 using the primers P35/80, the *clpP* gene full length with its own promoter was amplified with the primer pairs P2525/2526 (Supplementary Table 2). The plasmid was constructed with LIC and the resulting plasmid, p*clpP*, was electroporated into *S. aureus* strain RN4220 and then into NMΔ*clpP* with Φ85.

To express Sle1 proteins in *S. aureus*, the gene *sle1* was PCR-amplified with the following primer pairs: PL96/97 (Supplementary Table 2). The amplified fragments were digested with either *BamHI/SalI*. Then they were inserted into the multi-copy plasmid pOS1 under the control of its own promoter, resulting in pOS1-Sle1-his. The plasmids were transformed into *E. coli* DH5a, and then into *S. aureus* RN4220. Finally, the plasmids were transduced by Φ85 into wild type and *clpP* transposon mutants of *S. aureus*. The test strains in TSB containing chloramphicol (10 μg/ml) were incubated until early stationary growth phase (OD_600_ is around 2). The proteins were detected by Western blot analysis with anti-His-tag antibody.

### DNA manipulation

Unless stated otherwise, all restriction enzymes and DNA modification enzymes were purchased from New England Biolabs. Plasmids and genomic DNA were extracted with plasmid miniprep kit (Omega) according to the manufacturer's instruction. Plasmid DNA was introduced into *E. coli* by the method of Hanahan (Hanahan, 1983) and into *S. aureus* RN4220 by electroporation with a gene pulser (Bio-Rad). Subsequent transduction of the plasmids into target strains of *S. aureus* was carried out with Φ 85.

### Real-time quantitative reverse transcription-PCR (RT-PCR)

Cells in 3 ml TSB cultures were harvested at the stationary phase (OD_600_ value of 2). Cells were disrupted by shaking with a Mini-Beadbeater (Biospec Products) at maximum speed for 30 s. Tubes were then incubated on ice for 5 min. Then, the suspension was centrifuged. Total RNA isolation from the supernatant was performed according to the manufacturer's instructions (Qiagen). After treatment using a TURBO DNA-freeTM kit (Ambion), ~1 μg of total RNA was reverse-transcribed with a HieffTM first Strand cDNA Synthesis Super Mix for RT-PCR kit (Yeasen Bio). The cDNA was used as a template for real-time PCR using SYBR-green PCR reagents (Roche). Reactions were performed in a MicroAmp Optical 96-well reaction plate using a 7,500 Sequence Detector (Applied Biosystems). Primers used are listed in Supplementary Table 2. All RT-PCR experiments were performed in triplicate, with gyrase B (*gyrB*) used as an internal control.

### Western blot hybridization

Western blot analysis of proteins was carried out as described previously (Sun et al., 2011). The AgrA antibody was generated by GLbiochem, China. The His-tag antibody was bought from Yeasen Bio, China. All other antibodies were generated by GenScript.

### Semi-quantitative biofilm assay

Semi-quantitative biofilm assays were performed as described in our previous work (Liu et al., 2011). Briefly, overnight cultures of *S. aureus* strains were diluted 1:100 into fresh TSB with 0.5% Glucose. The diluted cultures were pipetted into sterile 96-well flat-bottom tissue culture plates and incubated at 37°C for 24 h. Culture supernatants were gently removed, and wells were washed with phosphate-buffered saline (PBS). The adherent organisms at the bottom of the wells were fixed by Bouin fixative over 1 h. Then the fixative was gently removed, wells were washed with PBS and stained with 0.4% (wt/vol) crystal violet. Biofilm formation was measured with a MicroELISA autoreader.

### Biofilm formation using a BioFlux microfluidic flow cell system

The BioFlux 1000z microfluidic system (Fluxion Biosciences, CA) was used to assess biofilm formation under flow conditions as described (Benoit et al., 2010). To grow biofilms, the microfluidic channels were primed with TSB supplemented with 0.5% glucose at 2 dynes/cm^2^ for 10 min. Each channel of a 48-well plate was coated with 20% platelet-poor human plasma in 50 mM carbonate buffer, PH 9.6 and incubated for 24 h at 4°C before biofilm assays were set-up. The exponential cultures were diluted 1:100 in TSB supplemented with 0.5% glucose. Bacterial suspensions were seeded at 0.2 dyn/cm^2^ for 5 s in all channels. The plate was then incubated at room temperature for 1 h to allow cells to adhere. Excess inoculums were removed and 0.8 ml of TSB supplemented with 0.5% glucose was added to the input wells. Biofilms were grown at 37°C with a flow of fresh media at a constant shear of 0.15 dyn/cm^2^. Images were taken every 10 min for 24 h at 10 × magnification under brightfield.

### Extracellular (e) DNA in culture supernatants

Cell cultures were grown to exponential phase. Culture supernatants from strain wild-type and its derivatives were filter sterilized, extracted with phenol-chloroform, and ethanol precipitated. Precipitates were suspended in H_2_O. The volumes of water were varied to compensate for slight differences in OD_600_ of the initial cultures. The eDNAs were quantified using Nanodrop 2000 (Thermo Scientific) and visualized by agarose gel.

### Triton X-100 induced autolysis assays

Bacterial cells were grown in TSB containing 1 M NaCl to the exponential growth phase at 37°C with shaking and then washed and suspended in 0.05 M Tris-HCl buffer (pH 7.5) containing 0.1% Triton X-100 and were incubated at 30°C with shaking. The optical density was measured in intervals. Results were normalized to an OD_600_ at time zero.

### Zymographic analysis

Cell cultures were grown to exponential phase, and normalized by OD_600_. The normalized supernatants were filtered through 0.22 μM filter and concentrated with a Centricon 3 concentrator (Millipore) at 4°C according to the manufacturer's instructions. Concentrated supernatants were mixed with an equal volume of Laemmli sample buffer, and 20 μl was separated by 12% SDS-PAGE supplemented with 0.2% autoclaved *S. aureus* RN4220 cell (wt/vol) as a substrate. After electrophoresis, the gels were washed four times with distilled water for 30 min at room temperature, incubated in 25 mM Tris-HCl containing 1.25% Triton X-100 (pH 8.0) at 37°C for 4 h, and then stained with methylene blue in 0.01% KOH(wt/vol), followed by destaining in deionized water.

### Animal infection model

A murine subcutaneous foreign body infection model was performed as described by Pozzi et al. (2012). Briefly, 20 male BALB/c mice were randomly and evenly divided into two groups and infected with wild type or *clpP* mutant bacteria. One-centimeter long silicon catheters (14 gauge) were implanted subcutaneously on each side at the flank of each mouse. Bacterial cells were grown in TSB to the exponential growth phase and then washed with and suspended in phosphate buffered saline (PBS) to OD_600_ = 1.0. *S. aureus* (10^8^ CFU) were injected into the catheter bed. After 7 days, the mice were sacrificed. Catheters were aseptically removed and manipulated with the method described previously except with a sonication time of 15 min (Liu et al., 2011). Furthermore, peri-catheter tissue, liver, spleen, kidneys were dissected, weighed, and homogenized in 1 ml PBS. Then serial dilutions of the wash fluid were plated on TSA blood plates, and the recovered *S. aureus* colonies were counted. Bacteria present in blood were also quantitatively cultured on TSA blood plates for CFU counting.

### Statistics

Statistical analysis was performed using Graph-Pad Prism, version 5. Error bars in all graphs show the standard deviation (± SD).

## Results

### ClpP, but not its ATPases, inhibits biofilm formation in *Staphylococcus aureus*

**Figure 1:**
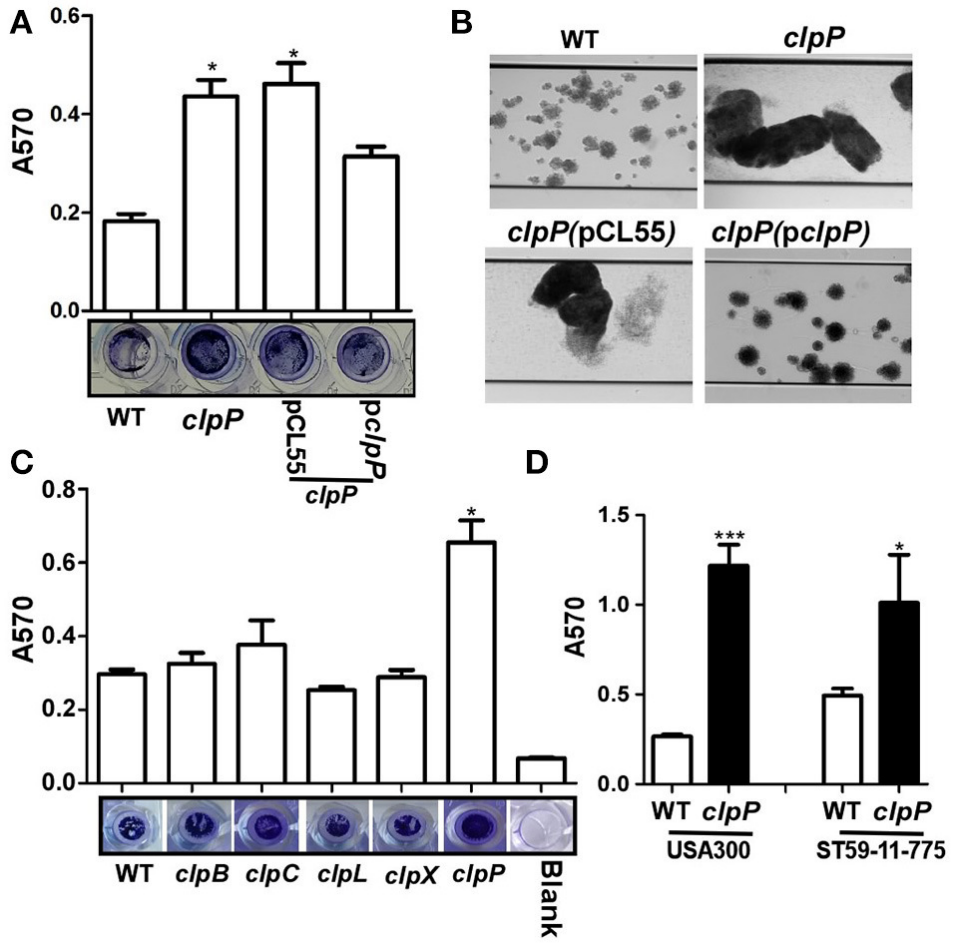
**ClpP, but not its ATPases, inhibits biofilm formation in ***S. aureus***. (A)** Biofilm formation was determined in *clpP* mutant and complement strains in comparison to Newman (WT) *in vitro* by semi-quantitative biofilm assay. The density of crystal violet-stained biofilms was measured at A_570_. Data were derived from three independent experiments, with four replicates of each experiment. ^*^p < 0.05 (vs. WT). The bottom showed crystal staining for biofilm formation. **(B)** Newman (WT), *clpP* mutant and complement strains were grown in TSB supplemented with 0.5% glucose in flow cells pre-coated with human plasma at 0.15 dynes/cm^2^ shear using a Biofulx 1000z instrument. Brightfield images depicting biofilm accumulation after 24 h were captured at 10 × magnification and are representative of at least three independent experiments. **(C)** ClpP chaperons are not involved in biofilm formation individually. Biofilm formation was determined in *clpB, clpC, clpL, clpX*, and *clpP* mutant in comparison to Newman (WT) *in vitro* by semi-quantitative biofilm assay. The density of crystal violet-stained biofilms was measured at A_570_. Data were derived from three independent experiments, with four replicates of each experiment. ^*^p < 0.05 (vs. WT). The bottom showed crystal staining for biofilm formation. **(D)** Biofilm formation was determined in *clpP* mutant in comparison to USA300 and ST59-11-775 WT strains *in vitro* by semi-quantitative biofilm assay. The density of crystal violet-stained biofilms was measured at A_570_. Data were derived from three independent experiments, with four replicates in each experiment. ^*^P < 0.05; ^***^P < 0.001 (vs. WT).

ClpP has been reported to inhibit biofilm formation in the *S. aureus* 8325-4 strain (Frees et al., 2004). To examine whether the biofilm inhibitory effect is universal, we analyzed biofilm formation of *clpP* mutant in the following three strains: *S*. *aureus* Newman, a methicillin-sensitive strain (MSSA), USA300, the predominant community-associated methicillin-resistant strain (CA-MRSA) in the USA and ST59 11-775, the most frequent lineage of community-associated infections in China and adjacent Asian countries (Li et al., 2016). In all three strains, *clpP* mutants showed enhanced biofilm formation (Figure 1), indicating that the biofilm inhibitory effect of ClpP is likely to be universal among *S. aureus* strains. When biofilm formation was analyzed in the presence of shear stress (0.15 dynes/cm^2^), which imitates blood flow in veins, the biofilm inhibitory effect of ClpP was still observed (Figure 1B). On the other hand, the mutants of Clp ATPases showed a wild-type level of biofilm formation (Figure 1C), indicating that either the Clp ATPases are not involved in the regulation of the biofilm formation or they have redundant roles.

### Proteins and eDNA are the main components of the biofilm formed by *clpP* mutant

**Figure 2:**
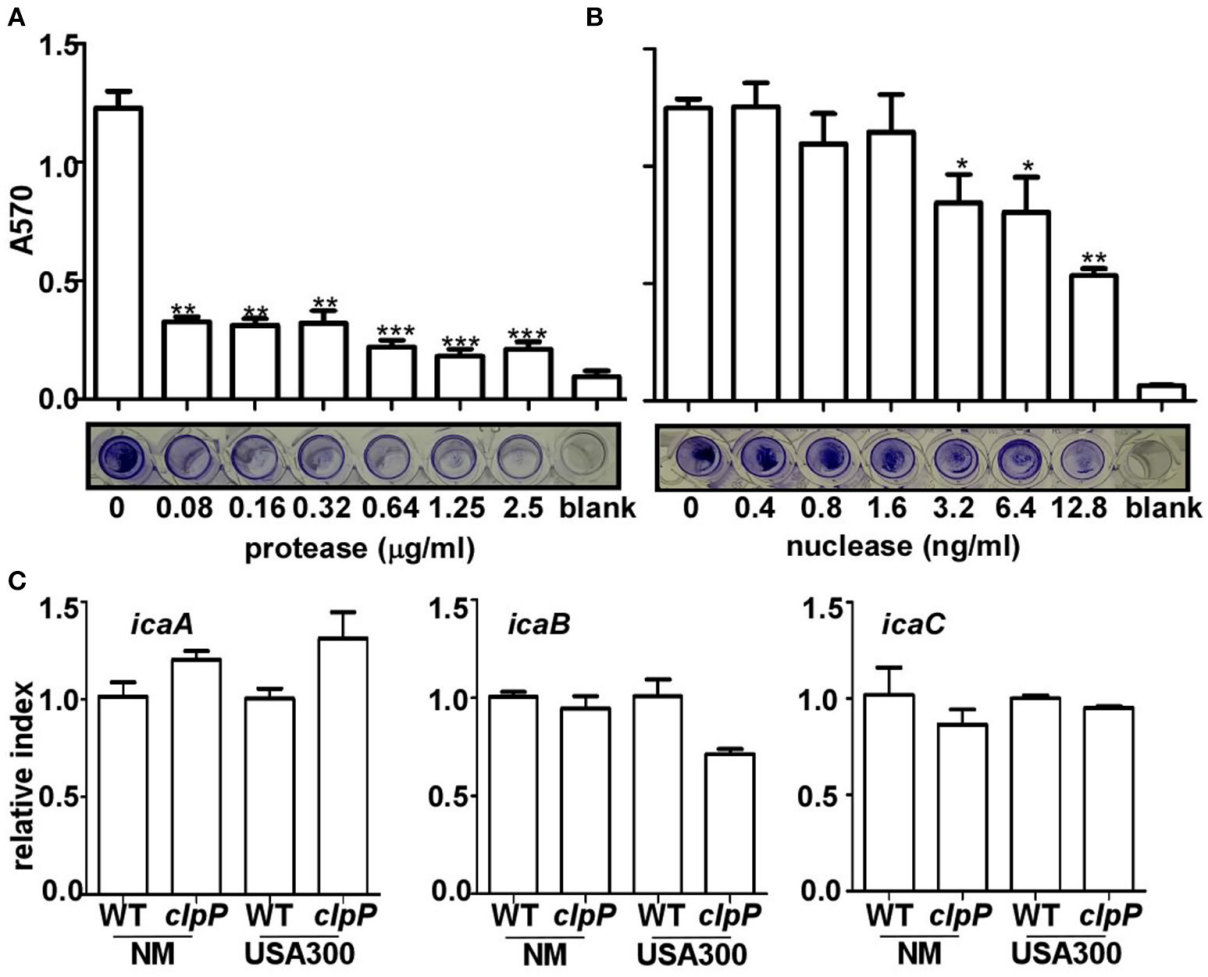
**Proteins and eDNA, but not PIA, are involved in biofilm formation by ***clpP*** mutant**. Degradation of *clpP* mutant biofilms by various concentrations of protease **(A)** and nuclease **(B)**. Biofilm formation by *clpP* mutant was determined *in vitro* by semi-quantitative biofilm assay. Data were from three independent experiments, with four replicates of each experiment. ^*^P < 0.05; ^**^P < 0.01; ^***^P < 0.001 (vs. WT). The bottom showed crystal staining for biofilm formation. **(C)** The expression of the *icaA/icaB/icaC* genes were determined in samples prepared from stationary-phase (OD_600_ = 2) cells grown in TSB, using quantitative RT-PCR. The wild-type (WT) and *clpP* mutant (*clpP*) strains were used for the assay. Data were derived from three biological repeats.

In the staphylococcal biofilm, the three main components are proteins, extracellular DNA (eDNA) and PIA (Paharik and Horswill, 2016). To determine the main constituents of the biofilm formed by the *clpP* mutant, we first added Proteinase K or nuclease to the biofilm formed by the Newman *clpP* mutant. As shown in Figures 2A,B, the biofilm was reduced by either proteinase K or nuclease, indicating proteins and eDNA are the main components of the biofilm formed by the *clpP* mutant.

The involvement of PIA in the biofilm formation by the *clpP* mutant was assessed indirectly by comparing the transcriptional levels of *icaABC*, the genes encoding PIA-synthesis enzymes in wild type and *clpP* mutant. As shown in Figure 2C, the transcript levels of *icaABC* were not significantly affected by the *clpP* mutation, suggesting that PIA probably does not contribute to the increased biofilm formation by the *clpP* mutant.

### In Newman and USA300 strain backgrounds, the *clpP* mutation lowers the activity of Agr, resulting in reduced protease activity

**Figure 3:**
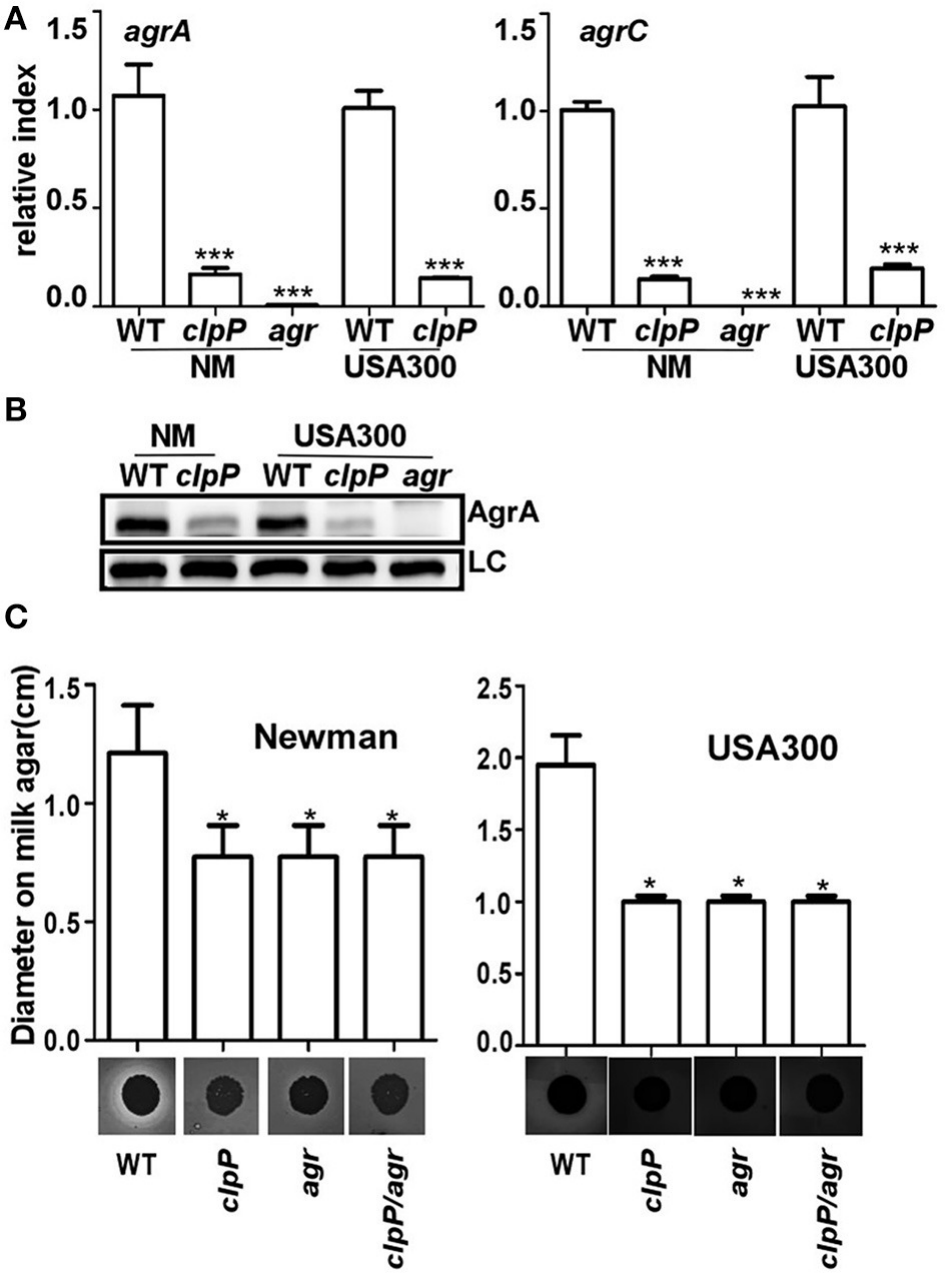
**In ***clpP*** mutant strain, protease activity is reduced due to the low Agr activity**. The transcription of the *agrA/agrC* genes **(A)** and the expression of AgrA **(B)** were determined in samples prepared from stationary-phase (OD_600_ = 2) cells grown in TSB using quantitative RT-PCR and Western blot. The wild-type (WT) and *clpP* mutant strains were used for the assay. *agr* mutant strain was used as a negative control. Data were derived from three biological repeats. ^***^P < 0.001 (vs. WT). LC, loading control. **(C)** Relative protease levels detected in wild type, *clpP, agr*, and *clpP/agr* double mutant strains grown in broth culture. Images show bacterial colonies and zones of clearing caused by protease activity on milk agar plates (20 g/L non-fat milk). ^*^P < 0.05 (vs. WT).

In *S. aureus*, the quorum sensing two component system Agr is known to suppress biofilm formation by up-regulating the production of extracellular proteases (Boles and Horswill, 2008). In the strain 8325-4, *clpP* mutation is known to reduce the activity of Agr (Frees et al., 2003; Michel et al., 2006). Indeed, the transcript levels of *agrA* and *agrC* were greatly reduced in the *clpP* mutants of both NM and USA300 strains (Figure 3A). The expression level of the response regulator AgrA was also greatly reduced in the *clpP* mutants (Figure 3B), further confirming the lower Agr activity in the *clpP* mutant. Finally, we observed that the protease activity was significantly decreased by mutation in either *clpP* or *agr* (Figure 3C), indicating that protease activity was reduced due to low Agr activity in *clpP* mutant strain.

### The overproduction of the cell wall hydrolase Sle1 is responsible for the increased release of eDNA

**Figure 4:**
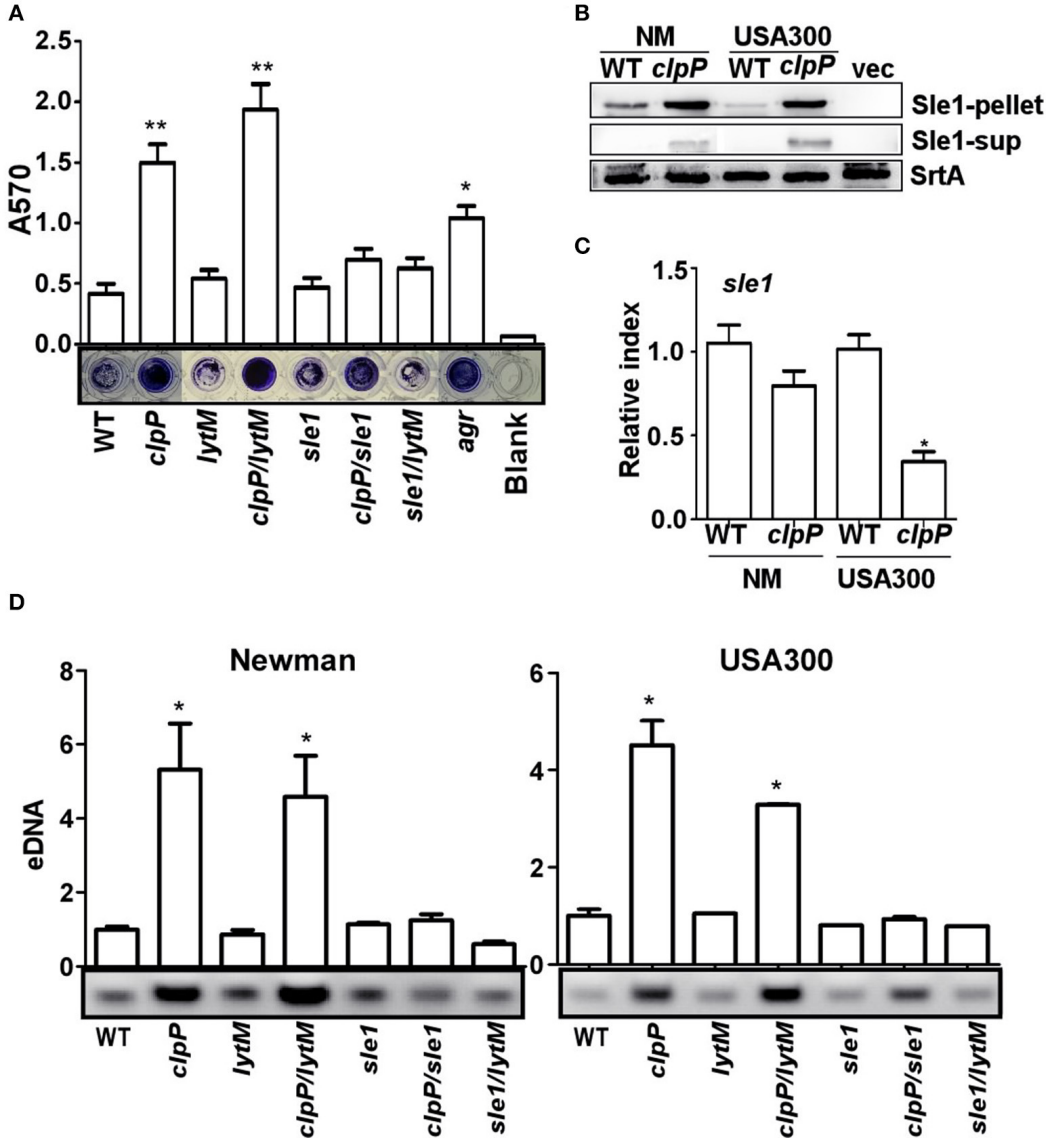
**The overproduction of the cell wall hydrolase Sle1 is responsible for the increased release of eDNA. (A)** Biofilm formation was determined in *sle1, lytM, clpP/sle1, clpP/lytM* double mutant strains in comparison to NM strain (WT) *in vitro* by semi-quantitative biofilm assay. *agr* mutant strain was used as a positive control. The density of crystal violet-stained biofilms was measured at 570 nm. Data were derived from three independent experiments, with four replicates of each experiment. ^*^P < 0.05; ^**^P < 0.01 (vs. WT). **(B)** The expression of the Sle1 protein was determined in samples prepared from stationary-phase (OD_600_ = 2) cells grown in TSB by Western blot. The wild-type (WT) and *clpP* mutant strains were used for the assay. Sle1-pellet, Sle1 protein detected in the cell pellets; Sle1-sup, Sle1 protein detected in the supernatant by TCA precipitation; SrtA, Sortase A was used as a loading control. **(C)** The expression of the *sle1* gene was determined in samples prepared from stationary-phase (OD_600_ = 2) cells grown in TSB, using quantitative RT-PCR. The wild-type (WT) and *clpP* mutant strains were used for the assay. Data were derived from three biological repeats. ^*^P < 0.05 (vs. WT). **(D)** Agarose gel of isolated eDNAs. Culture supernatants from the wild-type (WT) and its derivatives were filter sterilized, extracted with phenol-chloroform and ethanol precipitated. Precipitates were suspended in H_2_O normalized according to OD_600_ of the initial cultures. Data were derived from three biological repeats. ^*^P < 0.05(vs. WT).

Next we decided to determine the origin of the eDNA in the biofilm of the *clpP* mutant (Figure 2B). Since the release of eDNA is likely due to autolysis of *S. aureus* cells, we focused on staphylococcal cell wall hydrolases. Among either proven or putative cell wall hydrolases in *S. aureus*, LytM and Sle1 are known to be affected by ClpP. The transcription of *lytM* was up-regulated in the *clpP* mutant of *S. aureus* 8325 strain (Michel et al., 2006), whereas, in the *S. aureus* strains 8325-4 and Newman, Sle1 was shown to bind to ClpPtrap, the protease-deficient mutant of ClpP (Feng et al., 2013). Therefore, we examined whether either LytM or Sle1 play a role in the biofilm formation by the *clpP* mutant. As can be seen, while the deletion of *lytM* in the *clpP* mutant did not decrease the biofilm formation (*clpP/lytM* in Figure 4A), the *sle1* deletion did (*clpP/sle1* in Figure 4A), indicating that *sle1* is required for the biofilm formation by the *clpP* mutant. Indeed, a higher level of Sle1 was detected in the *clpP* mutant as compared with the wild type (Figure 4B). However, the transcription of *sle1* was either not significantly affected or reduced in the *clpP* mutants (Figure 4C). These results are consistent with the idea that the precursor of Sle1 is a ClpP substrate (Feng et al., 2013). Finally, to investigate whether the increased level of Sle1 leads to the release of more eDNA, we compared the amount of eDNA in culture supernatants of the test strains. Indeed, the culture supernatant of the *clpP* mutant contained a higher level of eDNA, as compared with that of wild type strain (*clpP* in Figure 4D). However, the level of eDNA was restored to that of wild type by further deletion of *sle1* (*clpP/sle1* in Figure 4D), strongly suggesting that the increased level of Sle1 is responsible for the heightened release of eDNA, leading to more robust biofilm formation of the *clpP* mutant.

### Sle1 is responsible for the increased autolysis of the *clpP* mutant

**Figure 5:**
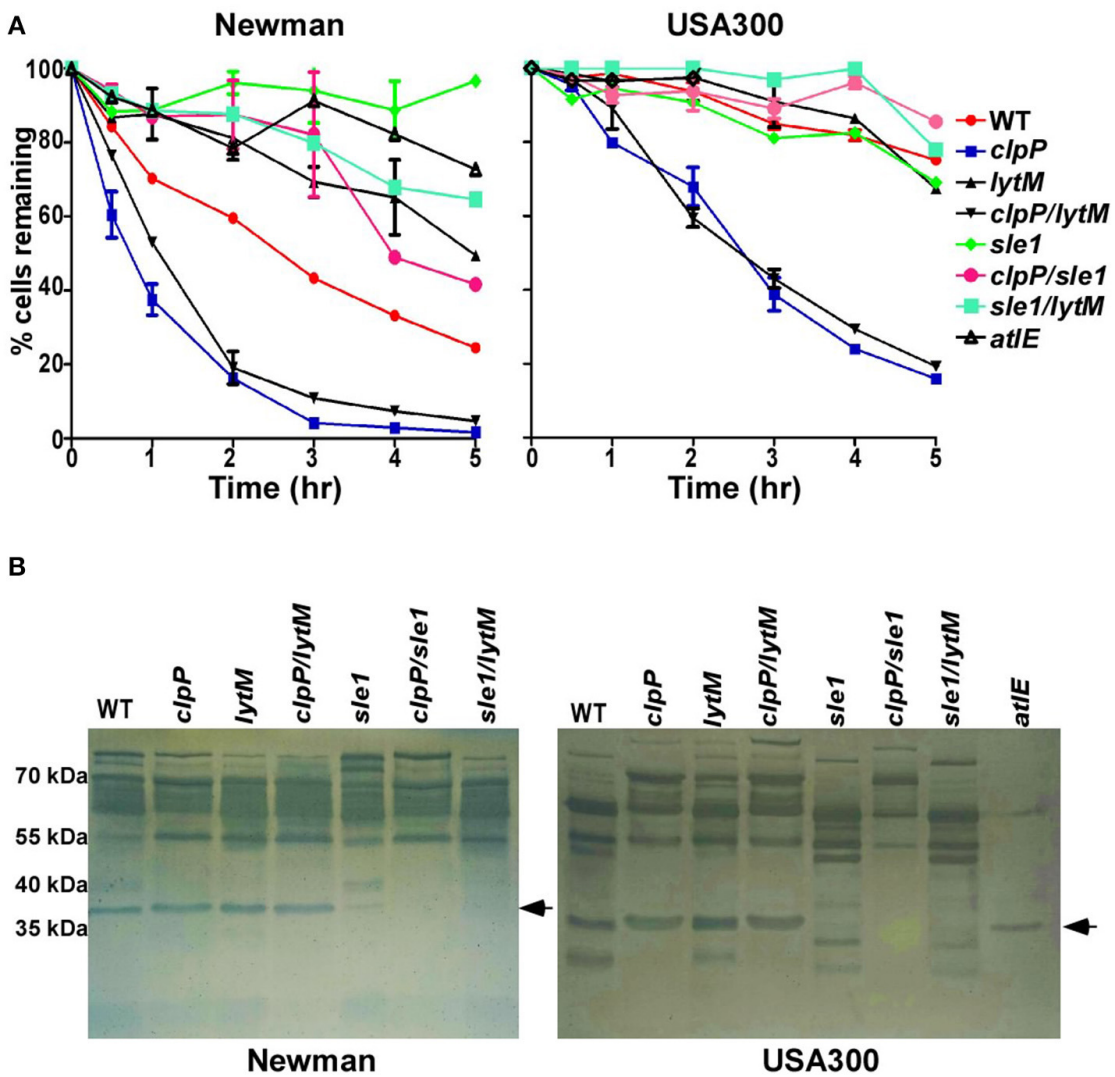
**Sle1 is responsible for the increase autolysis of the ***clpP*** mutant. (A)** Triton X-100 induced autolysis. The average of two independent experiments were shown. **(B)** Zymogram of *S. aureus* murein hydrolase activity. Concentrated culture supernatants from the wild-type (WT) and its derivatives were run on 12% acrylamide-SDS gels containing heat killed *S. aureus* RN4220 cells. Numbers to the left indicate the molecular weights of size standards. Dark bands indicate regions of murein hydrolase activity. The arrows showed the activity of murein hydrolase Sle1.

Sle1 is a peptidoglycan hydrolase, preferentially cleaving the N-acetylmuramyl-L-Ala bonds in dimeric cross-bridges that interlink the two murein strands in the peptidoglycan (Kajimura et al., 2005). Therefore, it is likely that the increased amount of eDNA in the culture supernatant of *clpP* mutant is due to the heightened cell lysis. To examine this notion, we measured autolysis activity of the test strains in the presence of Triton X-100. Indeed, the *clpP* mutation increased the autolysis activity of *S. aureus* Newman and USA300 (*clpP* in Figure 5A). Although the further deletion of *lytM* did not affect the autolysis activity of the *clpP* mutant (*clpP* vs. *clpP/lytM* in Figure 5A), the deletion of *sle1* greatly reduced the autolysis activity of the *clpP* mutant (*clpP* vs. *clpP/sle1* in Figure 5A). Zymography analysis showed that, in the test conditions, the cell wall hydrolase activities are mostly provided by the major autolysin Atl and Sle1, while the *clpP* mutant produced a higher level of Atl compared to WT only in USA300 strain. However, the *clpP* mutation specifically increases the overall activity of Sle1 (*clpP* vs. *clpP/sle1* in Figure 5B).

### The role of *clpP* in the pathogenesis of *S. aureus* biofilm-associated infection

**Figure 6:**
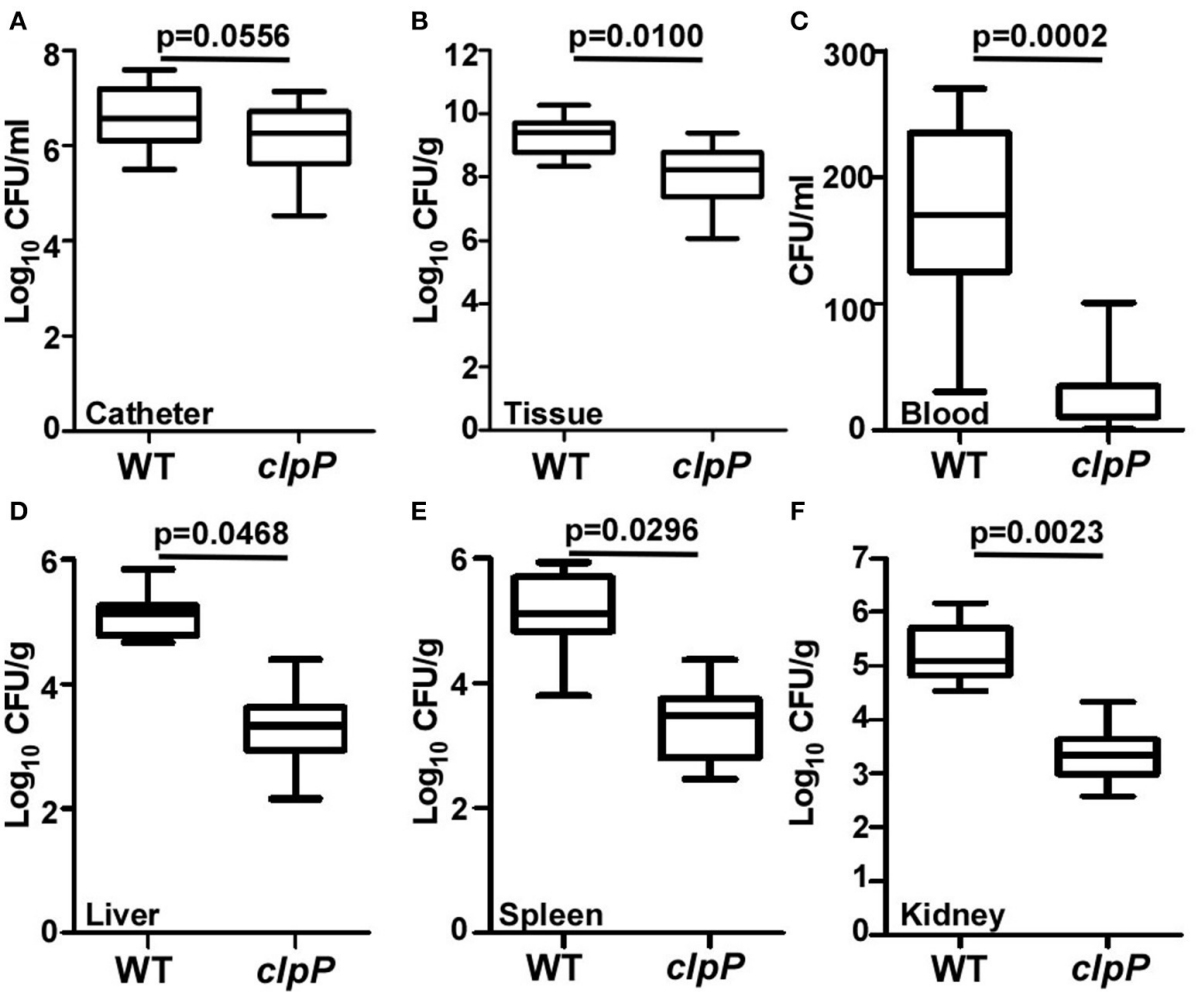
**Catheter colonization and dissemination of NM and ***clpP*** mutant strains in a mouse device-related infection model**. Implanted catheter segments were injected with 1 × 10^8^ bacteria and mice were sacrificed after 7 days. Colony forming units (CFU) per catheter **(A)**, per g peri-catheter tissue **(B)**, per ml blood **(C)**, per g liver **(D)**, per g spleen **(E)**, per g kidney **(F)** recovered from animals infected with wild type (NM) and *clpP* mutant strain. Statistical significance (*p-*values) is indicated in the figure.

ClpP is required for pathogenesis in a systemic staphylococcal infection as well as skin abscess infection (Frees et al., 2003; Farrand et al., 2013), indicating that ClpP plays important roles in *S. aureus* pathogenesis. Although the *clpP* mutant showed a slight growth defect especially during early exponential phase, the final cell density was similar to that of wild-type at lag phase (Supplementary Figure 1). Because *clpP* mutant showed an increased biofilm formation, we asked whether the increased biofilm formation can compensate the important virulence contributions of ClpP. A murine model of subcutaneous foreign body infection was used to investigate the impact of *clpP* on biofilm-associated infection *in vivo*. Progression of disease was measured by determining bacterial loads on implanted catheters while dissemination of the bacteria was examined by detecting bacterial loads on peri-tissues and organs. Wild type and the *clpP* mutant showed similar bacterial loads in the infected animals (Figure 6A). However, significantly higher number of bacteria were found in peri-catheter tissue as well as blood, liver, spleen, and kidneys in the animals infected by the wild-type strain, as compared with the animals infected by the *clpP* mutant strain (Figures 6B–F), indicating that ClpP plays a critical role in the dissemination of *S. aureus*, and that the increased biofilm formation capability cannot compensate the critical role played by ClpP.

## Discussion

In *S. aureus*, biofilm is an important virulence determinant in hospital-associated infections on indwelling medical devices (Joo and Otto, 2012). Staphylococcal biofilm formation is known to be controlled by multiple global regulators including ClpP, the proteolytic component of the Clp proteolysis system. However, it has not been elucidated how ClpP controls biofilm formation (Frees et al., 2004). In this study, we provided evidence that ClpP controls staphylococcal biofilm formation by activating the Agr quorum sensing system, a negative regulator of biofilm formation, and suppressing the expression of the murein hydrolase Sle1 at a post-transcriptional level. Moreover, using a murine model of subcutaneous foreign body infection, we also demonstrated that, in the *clpP* mutant, the increased biofilm formation cannot compensate the other critical ClpP functions missing in the mutant.

The Agr quorum sensing system plays important roles in staphylococcal virulence by suppressing the expression of surface protein expression and activating the expression of secreted proteins such as hemolysin and enzymes (Le and Otto, 2015). In particular, the Agr system is known to promote the detachment of biofilm by inducing the expression of the metalloprotease Aur and the serine proteases SplABCDEF (Boles and Horswill, 2008). Therefore, it is likely that the reduced expression of those proteins contributed to the decreased protease activity (Figure 3C) and the increased biofilm formation (Figure 1). Then how does ClpP activate the activity of Agr? ClpP is a protease, and the degradation of either AgrA or AgrC by ClpP would result in reduced, not increased, activity of Agr. Therefore, it is likely that ClpP controls Agr indirectly, possibly by degradation of a negative regulator of Agr. For negative regulators of *agr* transcription, the DNA binding protein SarX and the two component systems ArlSR and SrrAB are known (Fournier et al., 2001; Yarwood et al., 2001; Manna and Cheung, 2006). It is possible that one of those negative regulators is degraded by ClpP. Further, work will need to address whether any of those or hitherto unknown regulators are involved in the ClpP-mediated Agr activation.

Sle1 is a N-acetyl-muramyl-L-alanine amidase and plays an important role in splitting daughter cells during cell division (Kajimura et al., 2005). However, Sle1 does not appear to affect autolysis of staphylococcal cells (Kajimura et al., 2005). Indeed, in our study, deletion of *sle1* did not significantly affect autolysis, eDNA release, or biofilm formation (WT vs. *sle1* in Figures 4, 5A). In contrast, in *clpP* mutant background, the deletion of *sle1* abolished autolysis, eDNA release, and biofilm formation (*clpP* vs. *clpP/sle1* in Figures 4, 5A). It is likely that, in the wild type cells, ClpP-mediated control of Sle1 limits the Sle1 function only to daughter cell cleavage (Figure 4B). However, in the *clpP* mutant, due to increased concentration (Figure 4B), Sle1 seems to play not only in daughter cell separation, but also autolysis of the cells (Figure 5A). Unlike Sle1, LytM did not play any role in autolysis, eDNA release, or biofilm formation regardless of the mutation in *clpP* (*lytM* and *clpP/lytM* in Figures 4, 5A). LytM was initially suggested to be a glycylglycine endopeptidase (Ramadurai and Jayaswal, 1997). However, purified LytM did not show any lytic activity against *S. aureus* cells, and *lytM* deletion did not affect cell wall lysis (Singh et al., 2010). In our study, although the transcription of *lytM* was increased in the *clpP* mutant (Supplementary Figure 2), further deletion of *lytM* in the *clpP* mutant did not reduce biofilm formation or autolysis of the *clpP* mutant (Figures 4A, 5A), suggesting that LytM does not play a significant role in cell wall hydrolysis of *S. aureus*.

In a previous study, ClpX, an ATP-binding partner for ClpP, was shown to bind Sle1 (Feng et al., 2013). The inactivation of the ClpXP protease increased the β-lactam resistance by controlling cell wall metabolism through regulating Sle1 and Atl in JE2 strain (Baek et al., 2014). However, mutation in *clpX* did not significantly increase biofilm formation (Figure 1C), indicating that either Sle1 is delivered to ClpP by other ATPase partners or Sle1 overexpression alone is not sufficient to increase biofilm formation in the presence of functional Agr. On the other hand, although the mutation in *agr* greatly reduced protease production (Figure 3C), its effect on biofilm formation is not as significant as *clpP* mutation (Figure 4A), demonstrating the critical contribution of Sle1 to the biofilm formation. Since Sle1 is a secreted protein, it is likely that the precursor of Sle1 (i.e., Sle1 with signal peptide) binds to either ClpX or other Clp ATPase and is processed by ClpP.

**Figure 7:**
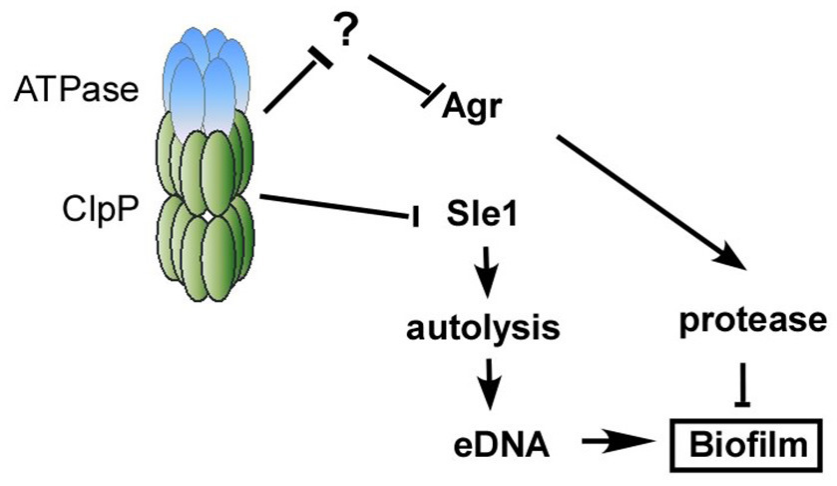
**Working model of the mechanism of how ClpP inhibits biofilm formation**. In a wild type condition, the Clp protease system is expected to degrade an unidentified negative regulator of Agr, resulting in Agr activation, which will limit the presence of proteins and repress biofilm formation. On the other hand, the Clp protease system degrades the precursor of cell wall hydrolase Sle1, resulting in the suppression of cell autolysis. The suppression of autolysis will decrease the release of eDNA and repress the formation of staphylococcal biofilm.

Figure 7 illustrates our current model of how ClpP represses biofilm formation in *S. aureus*. In a wild type condition, the Clp protease system is expected to degrade an unidentified negative regulator of Agr and Sle1, resulting in Agr activation and suppression of cell autolysis. The activated Agr and suppression of autolysis will limit the presence of proteins and eDNA and repress the formation of staphylococcal biofilm.

## Author contributions

TB, QL, ML, and XL conceived the study. QL, XW, JQ, SC, W-SY, LH, and XM performed experiments. QL, XW, TB, ML, and XL analyzed the data. TB, QL, ML, and XL drafted the manuscript. QL, XW, JQ, XL, ML, and TB revised and approved the manuscript.

### Conflict of interest statement

The authors declare that the research was conducted in the absence of any commercial or financial relationships that could be construed as a potential conflict of interest.

## References

[B1] Arnold I. Langer T. (2002). Membrane protein degradation by AAA proteases in mitochondria. Biochim. Biophys. Acta 1592, 89–96. 10.1016/S0167-4889(02)00267-7 12191771

[B2] Aslanidis C. De Jong P. J. (1990). Ligation-independent cloning of PCR products (LIC-PCR). Nucleic Acids Res. 18, 6069–6074. 10.1093/nar/18.20.6069 2235490 PMC332407

[B3] Bae T. Schneewind O. (2006). Allelic replacement in *Staphylococcus aureus* with inducible counter-selection. Plasmid 55, 58–63. 10.1016/j.plasmid.2005.05.005 16051359

[B4] Baek K. T. Grundling A. Mogensen R. G. Thogersen L. Petersen A. Paulander W.. (2014). β-lactam resistance in methicillin-resistant *Staphylococcus aureus* USA300 is increased by inactivation of the ClpXP protease. Antimicrob. Agents Chemother. 58, 4593–4603. 10.1128/AAC.02802-14 24867990 PMC4136064

[B5] Benoit M. R. Conant C. G. Ionescu-Zanetti C. Schwartz M. Matin A. (2010). New device for high-throughput viability screening of flow biofilms. Appl. Environ. Microbiol. 76, 4136–4142. 10.1128/AEM.03065-09 20435763 PMC2897429

[B6] Boles B. R. Horswill A. R. (2008). Agr-mediated dispersal of *Staphylococcus aureus* biofilms. PLoS Pathog. 4:e1000052. 10.1371/journal.ppat.1000052 18437240 PMC2329812

[B7] Chen C. Krishnan V. Macon K. Manne K. Narayana S. V. Schneewind O. (2013). Secreted proteases control autolysin-mediated biofilm growth of *Staphylococcus aureus*. J. Biol. Chem. 288, 29440–29452. 10.1074/jbc.M113.502039 23970550 PMC3795244

[B8] Cramton S. E. Gerke C. Schnell N. F. Nichols W. W. Gotz F. (1999). The intercellular adhesion (ica) locus is present in *Staphylococcus aureus* and is required for biofilm formation. Infect. Immun. 67, 5427–5433. 10496925 10.1128/iai.67.10.5427-5433.1999 PMC96900

[B9] Dalbey R. E. Wang P. Van Dijl J. M. (2012). Membrane proteases in the bacterial protein secretion and quality control pathway. Microbiol. Mol. Biol. Rev. 76, 311–330. 10.1128/MMBR.05019-11 22688815 PMC3372248

[B10] Farrand A. J. Reniere M. L. Ingmer H. Frees D. Skaar E. P. (2013). Regulation of host hemoglobin binding by the *Staphylococcus aureus* Clp proteolytic system. J. Bacteriol. 195, 5041–5050. 10.1128/JB.00505-13 23995637 PMC3811588

[B11] Feng J. Michalik S. Varming A. N. Andersen J. H. Albrecht D. Jelsbak L.. (2013). Trapping and proteomic identification of cellular substrates of the ClpP protease in *Staphylococcus aureus*. J. Proteome Res. 12, 547–558. 10.1021/pr300394r 23253041

[B12] Fournier B. Klier A. Rapoport G. (2001). The two-component system ArlS-ArlR is a regulator of virulence gene expression in *Staphylococcus aureus*. Mol. Microbiol. 41, 247–261. 10.1046/j.1365-2958.2001.02515.x 11454217

[B13] Frees D. Chastanet A. Qazi S. Sorensen K. Hill P. Msadek T.. (2004). Clp ATPases are required for stress tolerance, intracellular replication and biofilm formation in *Staphylococcus aureus*. Mol. Microbiol. 54, 1445–1462. 10.1111/j.1365-2958.2004.04368.x 15554981

[B14] Frees D. Qazi S. N. Hill P. J. Ingmer H. (2003). Alternative roles of ClpX and ClpP in *Staphylococcus aureus* stress tolerance and virulence. Mol. Microbiol. 48, 1565–1578. 10.1046/j.1365-2958.2003.03524.x 12791139

[B15] Gotz F. (2002). Staphylococcus and biofilms. Mol. Microbiol. 43, 1367–1378. 10.1046/j.1365-2958.2002.02827.x 11952892

[B16] Hanahan D. (1983). Studies on transformation of *Escherichia coli* with plasmids. J. Mol. Biol. 166, 557–580. 10.1016/S0022-2836(83)80284-8 6345791

[B17] Jefferson K. K. Pier D. B. Goldmann D. A. Pier G. B. (2004). The teicoplanin-associated locus regulator (TcaR) and the intercellular adhesin locus regulator (IcaR) are transcriptional inhibitors of the ica locus in *Staphylococcus aureus*. J. Bacteriol. 186, 2449–2456. 10.1128/JB.186.8.2449-2456.2004 15060048 PMC412131

[B18] Joo H. S. Otto M. (2012). Molecular basis of *in vivo* biofilm formation by bacterial pathogens. Chem. Biol. 19, 1503–1513. 10.1016/j.chembiol.2012.10.022 23261595 PMC3530155

[B19] Kajimura J. Fujiwara T. Yamada S. Suzawa Y. Nishida T. Oyamada Y.. (2005). Identification and molecular characterization of an N-acetylmuramyl-L-alanine amidase Sle1 involved in cell separation of *Staphylococcus aureus*. Mol. Microbiol. 58, 1087–1101. 10.1111/j.1365-2958.2005.04881.x 16262792

[B20] Kiedrowski M. R. Kavanaugh J. S. Malone C. L. Mootz J. M. Voyich J. M. Smeltzer M. S.. (2011). Nuclease modulates biofilm formation in community-associated methicillin-resistant *Staphylococcus aureus*. PLoS ONE 6:e26714. 10.1371/journal.pone.0026714 22096493 PMC3214024

[B21] Lauderdale K. J. Boles B. R. Cheung A. L. Horswill A. R. (2009). Interconnections between Sigma, B, *agr*, and proteolytic activity in *Staphylococcus aureus* biofilm maturation. Infect. Immun. 77, 1623–1635. 10.1128/IAI.01036-08 19188357 PMC2663138

[B22] Le K. Y. Otto M. (2015). Quorum-sensing regulation in staphylococci-an overview. Front. Microbiol. 6:1174. 10.3389/fmicb.2015.01174 26579084 PMC4621875

[B23] Li M. Wang Y. Zhu Y. Dai Y. Hong X. Liu Q.. (2016). Increased community-associated infections caused by panton-valentine leukocidin-negative MRSA, Shanghai, 2005-2014. Emerg. Infect. Dis. 22, 1988–1991. 10.3201/eid2211.160587 27767912 PMC5088022

[B24] Li T. He L. Song Y. Villaruz A. E. Joo H. S. Liu Q.. (2015). AraC-type regulator Rsp adapts *Staphylococcus aureus* gene expression to acute infection. Infect. Immun. 84, 723–734. 10.1128/IAI.01088-15 26712209 PMC4771356

[B25] Liu Q. Fan J. Niu C. Wang D. Wang J. Wang X.. (2011). The eukaryotic-type serine/threonine protein kinase Stk is required for biofilm formation and virulence in *Staphylococcus epidermidis*. PLoS ONE 6:e25380. 10.1371/journal.pone.0025380 21966513 PMC3179523

[B26] Manna A. C. Cheung A. L. (2006). Expression of SarX, a negative regulator of agr and exoprotein synthesis, is activated by MgrA in *Staphylococcus aureus*. J. Bacteriol. 188, 4288–4299. 10.1128/JB.00297-06 16740935 PMC1482969

[B27] Marti M. Trotonda M. P. Tormo-Mas M. A. Vergara-Irigaray M. Cheung A. L. Lasa I.. (2010). Extracellular proteases inhibit protein-dependent biofilm formation in *Staphylococcus aureus*. Microbes Infect. 12, 55–64. 10.1016/j.micinf.2009.10.005 19883788

[B28] McCarthy H. Rudkin J. K. Black N. S. Gallagher L. O'Neill E. O'gara J. P. (2015). Methicillin resistance and the biofilm phenotype in *Staphylococcus aureus*. Front. Cell. Infect. Microbiol. 5:1. 10.3389/fcimb.2015.00001 25674541 PMC4309206

[B29] Merino N. Toledo-Arana A. Vergara-Irigaray M. Valle J. Solano C. Calvo E.. (2009). Protein A-mediated multicellular behavior in *Staphylococcus aureus*. J. Bacteriol. 191, 832–843. 10.1128/JB.01222-08 19047354 PMC2632097

[B30] Michel A. Agerer F. Hauck C. R. Herrmann M. Ullrich J. Hacker J.. (2006). Global regulatory impact of ClpP protease of *Staphylococcus aureus* on regulons involved in virulence, oxidative stress response, autolysis, and DNA repair. J. Bacteriol. 188, 5783–5796. 10.1128/JB.00074-06 16885446 PMC1540084

[B31] Mootz J. M. Benson M. A. Heim C. E. Crosby H. A. Kavanaugh J. S. Dunman P. M.. (2015). Rot is a key regulator of *Staphylococcus aureus* biofilm formation. Mol. Microbiol. 96, 388–404. 10.1111/mmi.12943 25612137 PMC4467170

[B32] Okshevsky M. Meyer R. L. (2015). The role of extracellular DNA in the establishment, maintenance and perpetuation of bacterial biofilms. Crit. Rev. Microbiol. 41, 341–352. 10.3109/1040841X.2013.841639 24303798

[B33] O'Neill E. Pozzi C. Houston P. Humphreys H. Robinson D. A. Loughman A. (2008). A novel *Staphylococcus aureus* biofilm phenotype mediated by the fibronectin-binding proteins, FnBPA and FnBPB. J. Bacteriol. 190, 3835–3850. 10.1128/JB.00167-08 18375547 PMC2395027

[B34] Otto M. (2013). Staphylococcal infections: mechanisms of biofilm maturation and detachment as critical determinants of pathogenicity. Annu. Rev. Med. 64, 175–188. 10.1146/annurev-med-042711-140023 22906361

[B35] Paharik A. E. Horswill A. R. (2016). The Staphylococcal biofilm: adhesins, regulation, and host response. Microbiol. Spectr. 4:VMBF-0022-2015 10.1128/microbiolspec.VMBF-0022-2015 PMC4887152 27227309

[B36] Pozzi C. Waters E. M. Rudkin J. K. Schaeffer C. R. Lohan A. J. Tong P.. (2012). Methicillin resistance alters the biofilm phenotype and attenuates virulence in *Staphylococcus aureus* device-associated infections. PLoS Pathog. 8:e1002626. 10.1371/journal.ppat.1002626 22496652 PMC3320603

[B37] Ramadurai L. Jayaswal R. K. (1997). Molecular cloning, sequencing, and expression of lytM, a unique autolytic gene of *Staphylococcus aureus*. J. Bacteriol. 179, 3625–3631. 10.1128/jb.179.11.3625-3631.1997 9171409 PMC179157

[B38] Rohde H. Burandt E. C. Siemssen N. Frommelt L. Burdelski C. Wurster S.. (2007). Polysaccharide intercellular adhesin or protein factors in biofilm accumulation of *Staphylococcus epidermidis* and *Staphylococcus aureus* isolated from prosthetic hip and knee joint infections. Biomaterials 28, 1711–1720. 10.1016/j.biomaterials.2006.11.046 17187854

[B39] Singh V. K. Carlos M. R. Singh K. (2010). Physiological significance of the peptidoglycan hydrolase, LytM, in *Staphylococcus aureus*. FEMS Microbiol. Lett. 311, 167–175. 10.1111/j.1574-6968.2010.02087.x 20738399 PMC2944916

[B40] Sun F. Cho H. Jeong D. W. Li C. He C. Bae T. (2011). Aureusimines in *Staphylococcus aureus* are not involved in virulence. PLoS ONE 5:e15703 10.1371/journal.pone.0015703 PMC3012096 21209955

[B41] Toledo-Arana A. Merino N. Vergara-Irigaray M. Debarbouille M. Penades J. R. Lasa I. (2005). *Staphylococcus aureus* develops an alternative, *ica*-independent biofilm in the absence of the *arlRS* two-component system. J. Bacteriol. 187, 5318–5329. 10.1128/JB.187.15.5318-5329.2005 16030226 PMC1196035

[B42] Yarwood J. M. McCormick J. K. Schlievert P. M. (2001). Identification of a novel two-component regulatory system that acts in global regulation of virulence factors of *Staphylococcus aureus*. J. Bacteriol. 183, 1113–1123. 10.1128/JB.183.4.1113-1123.2001 11157922 PMC94983

