# Supplementary material for: The ATP-Dependent Protease ClpP Inhibits Biofilm Formation by Regulating Agr and Cell Wall Hydrolase Sle1 in Staphylococcus aureus
Source: Front Cell Infect Microbiol. 2017 May 15;7:181. doi: 10.3389/fcimb.2017.00181 (PMC5430930; doi:10.3389/fcimb.2017.00181)
Supplement: Supplementary file 1 [file Table1.DOCX]

**Supplementary Table 1.**  **Bacterial strains and plasmids used in this study**

| **Strain or plasmid** | **Relevant characteristic** | **Origin or reference** |
| --- | --- | --- |
| ***E. coli*** |  |  |
| DH5α | Plasmid free, restriction deficient | New England Biolabs |
|  |  |  |
| ***S. aureus*** |  |  |
| RN4220 | Restriction deficient, prophage cured | ([Kreiswirth et al., 1983](#_ENREF_5)) |
| Newman | Clinical isolate, L18P substitution in SaeS | ([Duthie and Lorenz, 1952](#_ENREF_3)) |
| NMΔ*agr* | Newman with deletion of the *agr* operon | This study |
| NMΔ*sle1* | Newman with deletion of the *sle1* | This study |
| NMΔ*lytM* | Newman with deletion of the *lytM* | This study |
| USA300-P23 | USA300-0114 without plasmid 2 and 3 | ([Jeong et al., 2011](#_ENREF_4)) |
| USA300Δ*agr* | USA300-0114 with deletion of the *agr* | This study |
| USA300Δ*sle1* | USA300-0114 with deletion of the *sle1* | This study |
| USA300Δ*lytM* | USA300-0114 with deletion of the *lytM* | This study |
| 11-775 | ST59 clinical isolate | ([Li et al., 2016](#_ENREF_7)) |
| NE912 | Transposon mutant of *clpP* gene | NARSA |
| NE967 | Transposon mutant of *clpB* gene | NARSA |
| NE699 | Transposon mutant of *clpC* gene | NARSA |
| NE1219 | Transposon mutant of *clpL* gene | NARSA |
| NM*clpP* | Newman strain that acquired the *clpP* transposon mutation in NE912 via transduction |  |
| USA300*clpP* | USA300-P23 strain that acquired the *clpP* transposon mutation in NE912 via transduction |  |
| NM*clpB* | Newman strain that acquired the *clpB* transposon mutation in NE967 via transduction |  |
| NM*clpC* | Newman strain that acquired the *clpC* transposon mutation in NE699 via transduction |  |
| NM*clpL* | Newman strain that acquired the *clpL* transposon mutation in NE1219 via transduction |  |
| NM*clpX* | Newman strain that acquired the *clpX* transposon mutation using mariner transposon | This study |
| NE460 | Transposon mutant of *atlE* gene | NARSA |
| NM*atlE* | Newman strain that acquired the *atlE* transposon mutation in NE460 via transduction |  |
| USA300*atlE* | USA300-P23 strain that acquired the *atlE* transposon mutation in NE460 via transduction |  |
|  |  |  |
| ***Plasmid*** |  |  |
| pKOR1 | Allelic replacement plasmid | ([Bae and Schneewind, 2006](#_ENREF_1)) |
| pKOR1Δ*agr* | pKOR1 containing *agr* deletion cassette | This study |
| pKOR1Δ*sle1* | pKOR1 containing *sle1* deletion cassette | This study |
| pKOR1Δ*lytM* | pKOR1 containing *lytM* deletion cassette | This study |
| pCL55 | An integration vector for *S. aureus* | ([Lee et al., 1991](#_ENREF_6)) |
| pOS1 | An *E.coli- S. aureus* shuttle vector | ([Bubeck Wardenburg et al., 2006](#_ENREF_2)) |
| p*clpP* | pCL55 carrying the *clpP* gene with His-tag sequence at the C-terminus | This study |
| pOS1*-sle1*-his | pOS1 carrying *sle1* with His_6_-tag sequence | This study |

**REFERENCES**

Bae, T., and Schneewind, O. (2006). Allelic replacement in *Staphylococcus aureus* with inducible counter-selection. *Plasmid.* 55**,** 58-63.

Bubeck Wardenburg, J., Williams, W.A., and Missiakas, D. (2006). Host defenses against *Staphylococcus aureus* infection require recognition of bacterial lipoproteins. *Proc Natl Acad Sci U S A.* 103**,** 13831-13836.

Duthie, E.S., and Lorenz, L.L. (1952). Staphylococcal coagulase; mode of action and antigenicity. *J Gen Microbiol.* 6**,** 95-107.

Jeong, D.W., Cho, H., Lee, H., Li, C., Garza, J., Fried, M., and Bae, T. (2011). Identification of the P3 promoter and distinct roles of the two promoters of the SaeRS two-component system in *Staphylococcus aureus*. *J Bacteriol.* 193**,** 4672-4684.

Kreiswirth, B.N., Lofdahl, S., Betley, M.J., O'reilly, M., Schlievert, P.M., Bergdoll, M.S., and Novick, R.P. (1983). The toxic shock syndrome exotoxin structural gene is not detectably transmitted by a prophage. *Nature.* 305**,** 709-712.

Lee, C.Y., Buranen, S.L., and Ye, Z.H. (1991). Construction of single-copy integration vectors for *Staphylococcus aureus*. *Gene.* 103**,** 101-105.

Li, M., Wang, Y., Zhu, Y., Dai, Y., Hong, X., Liu, Q., Li, T., Qin, J., Ma, X., Lu, H., Xu, J., and Otto, M. (2016). Increased Community-Associated Infections Caused by Panton-Valentine Leukocidin-Negative MRSA, Shanghai, 2005-2014. *Emerg Infect Dis.* 22**,** 1988-1991.
