# Supplementary material for: The ATP-Dependent Protease ClpP Inhibits Biofilm Formation by Regulating Agr and Cell Wall Hydrolase Sle1 in Staphylococcus aureus
Source: Front Cell Infect Microbiol. 2017 May 15;7:181. doi: 10.3389/fcimb.2017.00181 (PMC5430930; doi:10.3389/fcimb.2017.00181)
Supplement: Supplementary file 2 [file Table2.DOCX]

**Supplementary Table 2. Oligonucleotides used in this study**

| **Name** | | **Sequence (5’ 🡪 3’)** | **Target** |
| --- | --- | --- | --- |
| P236 | ATTGGAAGTGGATAACGGTACCGGTTCCGAGGCTC | | LIC for pKOR1 |
| P237 | ATTGGATTGGAAGTACGGGCCCGAGCTTAAGACTGG | | LIC for pKOR1 |
| P596 | TACTTCCAATCCAATG GGCACAGGAACAGATATGGAAG | | For *lytM* deletion |
| P597 | GTATAAAACATCCTCCATTAAAG | | For *lytM* deletion |
| P598 | CTTTAATGGAGGATGTTTTATACTACAGAAAATCCCAAGTTGCGATATC | | For *lytM* deletion |
| P599 | TTATCCACTTCCAATG GTAGTACTTCATTATCGTTTAAACC | | For *lytM* deletion |
| P632 | TACTTCCAATCCAATG CTAAGGCTTGGGAGAAGGTTAAAG | | For *sle1* deletion |
| P633 | TTTAAAATCCTCCTCTTGCTTAAC | | For *sle1* deletion |
| P634 | GTTAAGCAAGAGGAGGATTTTAAAAGTCTTACGTATATAAATATATAATG | | For *sle1* deletion |
| P637 | TTATCCACTTCCAATG CATAATGTCGTAACATGATGGTGTC | | For *sle1* deletion |
| P19 | TACTTCCAATCCAATGTTTTAGTGTGAATAAAAGTGGCC | | For *agr* deletion |
| P20 | GATGAATAATTAATTACTTTCATTGTAAATTTG | | For *agr* deletion |
| P21 | CAATGAAAGTAATTAATTATTCATCACTTACCTATTTAACGTTTGTCTACAAAGTTG | | For *agr* deletion |
| P22 | TTATCCACTTCCAATGGTCACTGTAGTCATTTATACGAT | | For *agr* deletion |
| P35 | ATTGGAAGTGGATAACCGGAGGAGGGATGTAAAATGTGG | | LIC for pCL55 |
| P80 | ATTGGATTGGAAGTACGAATTCTTGAAGACGAAAGGGCCTCG | | LIC for pCL55 |
| P2525 | TTATCCACTTCCAATG GAATTGAACCCCTATCTTAAG | | p*clpP* |
| P2526 | TACTTCCAATCCAATG CTAATGATGATGATGATGATG TTTTGTTTCAGGTACCATCACTT | | p*clpP* |
| PL96 | GAG *GGATCC* GGTTAAAGATGGTGCTAAAGTTGTC | | Sle1 expression (Sa)^a^ |
| PL97 | GAG *GTCGAC* CTAATGATGATGATGATGATG GTGAATATATCTATAATTATTTACTTGG | | Sle1 expression (Sa)^a^ |
| PL98 | GTATCTGGCTCAAGTAATTCTAC | | Real-time PCR for *sle1* |
| PL99 | GCAGATCCTGAGTTCGTAGATGC | | Real-time PCR for *sle1* |
| P585 | GCAGGAGATAACAATGACTACAC | | Real-time PCR for *lytM* |
| P586 | TTACTTGCTGATCCACCATTTTG | | Real-time PCR for *lytM* |
| P404 | CGCTTGCCATGTGTTGGAT | | Real-time PCR for *icaA* |
| P405 | TCACGCGTTGCTTCCAAAG | | Real-time PCR for *icaA* |
| P78 | AGTCGCAGAAAACTATAGCCTATCCT | | Real-time PCR for *icaB* |
| P79 | CCGTATTTTAACCCAGCTTTTTTG | | Real-time PCR for *icaB* |
| P80 | GGGTGGATCCTTAGTGTTACAATTTT | | Real-time PCR for *icaC* |
| P81 | ACTTTTTGGTAATTCAAGGTTGTCAGT | | Real-time PCR for *icaC* |
| P82 | GCACATACACGCTTACAATTGTTG | | Real-time PCR for *agrA* |
| P83 | ACACTGAATTACTGCCACGTTTTAAT | | Real-time PCR for *agrA* |
| P84 | GCAAGTTCCGTCATGATTATGTCA | | Real-time PCR for *agrC* |
| P85 | GGCATGTCATCTTCTCGAATGTAT | | Real-time PCR for *agrC* |
| P43 | CAAATGATCACAGCATTTGGTACAG | | Real-time PCR for *gyrB* |
| P44 | CGGCATCAGTCATAATGACGAT | | Real-time PCR for *gyrB* |
