# Supplementary figures and images for: The ATP-Dependent Protease ClpP Inhibits Biofilm Formation by Regulating Agr and Cell Wall Hydrolase Sle1 in Staphylococcus aureus
Source: Front Cell Infect Microbiol. 2017 May 15;7:181. doi: 10.3389/fcimb.2017.00181 (PMC5430930; doi:10.3389/fcimb.2017.00181)

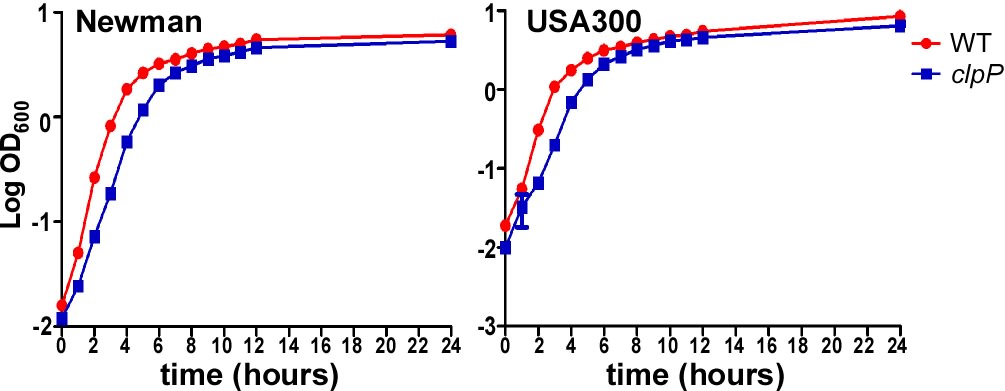

Supplement: Supplementary file 3 [file Image1.JPEG]

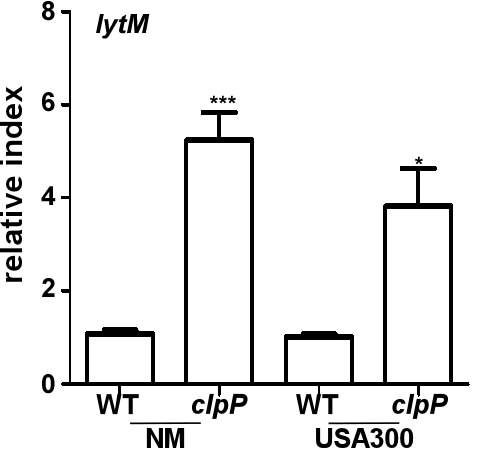

Supplement: Supplementary file 4 [file Image2.JPEG]
